# Proposal for a More Advantageous Method of Studying the Nature, Virtue, and Practical Application of Medicines, Founded on a Physiological Classification

**Published:** 1822-04

**Authors:** 


### MEDEOGRAPHY.

Art. X.—***Proposal for a more advantageous Method of studying the Nature, Virtue, and practical Application of Medicines, founded on a Physiological Classification.*** By the Editor.

THERE is, perhaps, no branch of medical science, for the study of which a greater number of methods and classifications have been proposed than Medeography. Some authors, like Manget, Ludolff, Geoffroy, Tessari, and Vogel, have considered a promiscuous description of the various medicines employed in practice perfectly sufficient for acquiring a knowledge of their virtue. Others have assumed the dose at which a remedy is administered, as the basis of their arrangement: among these we may reckon Boerhaave, Baldinger, and Lieutaud. A third class have limited themselves to an alphabetical distribution of medical substances, forming a sort of dictionary, to which the student might refer for information on each individual remedial agent. In this manner all the Dispensatories are compiled; while, in a fourth class of writers, we find the names of Linneus, Bergius, Murray, and others, as the advocates of a system of studying materia medica, in every respect similar to that which the illustrious Swede first proposed for the study of natural history.

Some think that Cullen's Materia Medica is the most judiciously arranged of any extant; but his divisions and subdivisions are not tenable. What assistance can it be to the student to know that, among the acrid substances, for instance, as Cullen has called them, there are fixed acrid substances, volatile acrid substances, and so on in succession.

Murray has arranged the articles of the materia medica according to the effects they are known to produce on the living system; but the *modus operandi* of medicines is often extremely obscure, and much theory must mingle with our ideas and language, in explaining nice distinctions between the sensible effects of remedial agents, if we adopt his system.

Alibert, who has published, by far, one of the best systems of Medeography, has followed a plan, in some respects, akin to that which we are about to propose to our readers; but, on comparing the two, some important variations will be found, which, in our own plan, appear to be calculated to facilitate the object in view. As it is with regard to their sensible effects on the animal economy, that a knowledge of the various substances of the materia medica is at all important, it seems a more natural division to consider them according to the particular physiological functions on which they may be supposed to exert their influence.

### PART I.—*Of Medicines in general, and their Mode of acting on the Animal System.*

A. Medicines acting specifically on the digestive organs.

1. Medicines calculated to correct certain morbid affections of the system, by their primary action on the stomach.

*a.* Permanent stimuli.

*α.* Tonics.

*β.* Astringents.

*b.* Diffusible stimuli.

*c.* Emetics.

*d.* Antacids.

i. From the vegetable }
ii. ———— mineral } kingdoms.
iii. ———— animal }

2. Medicines calculated to act on the system by their primary action on the intestinal canal.

*a.* Cathartics.

i. From the vegetable }
ii. ———— mineral } kingdoms.
iii. ———— animal }

3. Medicines directed to destroy, or counteract, the action of worms on the digestive organs.

*a.* Anthelmintics.

i. From the vegetable }
ii. ———— mineral } kingdoms.

4. Medicines calculated to prevent the development, or to correct the morbid effects of poisons on the digestive organs.

*a.* Antidotes—Alexipharmics.

i. From the vegetable }
ii. ———— mineral } kingdoms.

B. Medicines acting specifically on the respiratory system.

1. Medicines calculated to correct the morbid action of the respiratory organs from an accumulated secretion.

*a.* Expectorants.

i. From the vegetable }
ii. ———— mineral } kingdoms.
iii. ———— animal }

2. Application of certain gases, or aëriform fluids, to correct the morbid action of the respiratory organs from special causes.

*a.* Oxigene.
*b.* Hydrogene.
*c.* Atmospheric air, with an increased proportion of nitrogene.
*d.* Carbonic acid—oxide.
*e.* Nitrous oxide.

3. Application of certain aëriform fluids to the respiratory organs, to impede the action of contagion.

*a.* Chlorine.
*b.* Nitrous gas.
*c.* Sulphuretted hydrogen ?

4. Medicines directed to diminish the evolution of animal heat by the respiratory organs.

*a.* Refrigerants.
i. From the vegetable } kingdoms.
ii. ——— mineral }

C. Medicines acting specifically on the circulatory system.

1. Medicines intended to augment the energy of circulation.

§. General.
*a.* Analeptics.
*b.* Cordials.
i. From the vegetable } kingdoms.
ii. ——— mineral }
*c.* Excitement of certain passions.

§. Topical.
*a.* Electricity.
*b.* Baths.
*c.* Frictions.

2. Medicines intended to diminish the energy of the circulation.

§. General.
*a.* Deoxygenants.
*b.* Sedatives.
*c.* Contrastimulants ?
i. From the vegetable } kingdoms.
ii. ——— mineral }
*d.* Excitement of certain passions.

§. Topical.
*a.* External application of certain substances to the system.
*b.* Phlebotomy.
*c.* Scarifications.
*d.* Leeches.

3. Medicines calculated to direct or promote particular secretions, by their action on the circulatory system.

§. General.
*a.* Sialogues.
*b.* Diaphoretics.
*c.* Galactophora.
i. From the vegetable } kingdoms.
ii. ——— mineral }

§. Topical.

*a.* Errhines.

*b.* Epispastics.

*c.* Emollients.

i. From the vegetable ⎫ kingdoms.
ii. ———— mineral ⎭

4. Medicines directed to act immediately on the uterine system.

*a.* Emmenagogues.

i. From the animal ⎫
ii. ———— vegetable ⎬ kingdoms.
iii. ———— mineral ⎭

§. Topical.

D. Medicines intended to correct the morbidity of the urinary organs.

1. Medicines calculated to promote the secretion of urine.

*a.* Diuretics.

i. From the vegetable ⎫ kingdoms.
ii. ———— mineral ⎭

§. Topical.

2. Medicines calculated to alter the state of the urinary secretion.

*a.* Diluents.

*b.* Demulcents.

i. From the vegetable ⎫ kingdoms.
ii. ———— mineral ⎭

3. Medicines directed to dissolve urinary concretions.

*a.* Lithontriptics.

i. From the vegetable ⎫ kingdoms.
ii. ———— mineral ⎭

E. Medicines, the action of which is principally directed to the nervous system.

*a.* Narcotics.

*b.* Antispasmodics.

i. From the vegetable ⎫
ii. ———— mineral ⎬ kingdoms.
iii. ———— animal ⎭

F. Medicines intended for topical diseases.

PART II.—*On the Manner of distinguishing and knowing the Virtues of Medicines in general.*

A. Criterion of the medicinal action of pharmaceutic substances, derived from their taste.

1. Substances having a *sweet* taste.
2. ———— an *acrid* taste.
3. ———— an *oily* taste.
4. ———— an *astringent* taste.
5. ———— a *bitter* taste.
6. ———— an *insipid* taste.

7. Substances having a *nauseous* taste.
8. ———————— a *saltish* taste.
   a. Their medicinal virtues.

B. Criterion of the medicinal properties of pharmaceutic substances, derived from their smell.
1. Substances having a *camphorated* smell.
2. ———————— a *narcotic* smell.
3. ———————— an *æthereal* smell.
4. ———————— a *volatile acid* smell.
5. ———————— a *volatile alcaline* smell.
   a. Their medicinal virtues.

C. Of the mode of discovering, or ascertaining, the medicinal properties of new pharmaceutic substances.

D. Chemical analysis of vegetables.

Part III.—*Of Pharmaceutic Preparations.*

A. Simple preparations.
1. Conserves.
2. Juices.
3. Inspissated juices.
4. Fixed oils.
5. Emulsions and mixtures.
6. Infusions.
7. Mucilages.
8. Decoctions.
9. Syrups.

B. Complicated preparations.
1. Volatile oils.
2. Wines.
3. Vinegars.
4. Extracts.
5. Tinctures.
6. Distilled waters.
7. Distilled spirits.
8. Salts, and saline substances.
9. Prepared metallic substances.
10. Electuaries.
11. Ointments.

C. Mechanical preparations.
1. Powders.
2. Pills.
3. Troches.
4. Plasters and Cataplasms.

D. Analytical examination of mineral waters.

Part IV.—*On the Art of Writing Medical Formulæ, or the Prescribing the Composition of Medicines with elegance and correctness.*

